# The Ripple Effect of Climate Change: Assessing the Impacts on Water Quality and Hydrology in Addis Ababa City (Akaki Catchment)

**DOI:** 10.1155/2024/8824622

**Published:** 2024-01-17

**Authors:** Thandile T. Gule, Binyam Tesfaw Hailu, Brook Lemma

**Affiliations:** ^1^Africa Centre of Excellence for Water Management, Addis Ababa University, P.O. Box 1176, Addis Ababa, Ethiopia; ^2^Addis Ababa University, School of Earth Sciences, P.O. Box 1176, Addis Ababa, Ethiopia; ^3^Addis Ababa University, College of Natural and Computer Sciences, Department of Aquatic Sciences, P.O. Box 1176, Addis Ababa, Ethiopia

## Abstract

This research aimed to evaluate the effects of climate change on the hydrology and water quality in the Akaki catchment, which provides water to Addis Ababa, Ethiopia. This was performed using the soil and water assessment tool (SWAT) model and an ensemble of four global climate models under two Shared Socioeconomic Pathways (SSP) emission scenarios from Coupled Model Intercomparison Project Phase 6 (CMIP6). The climate data were downscaled and bias-corrected using the CMhyd tool and calibrated and validated using the SWAT-CUP software package. Change points and patterns in annual rainfall and temperature were determined using the homogeneity test and Mann–Kendell trend test. Water quality data were obtained from Addis Ababa Water and Sewerage Authority (AAWSA), and more samples were taken and analyzed in accordance with APHA recommended procedures. The SWAT model output was then used to assess the impacts of climate change on hydrological components and water quality. Rainfall increased by 19.39 mm/year under SSP2-4.5 and 12.8 mm/year under SSP8.5. Maximum temperature increased by 0.03°C/yr for SSP2-4.5 and 0.04°C/yr for SSP5-8.5. Minimum temperature increased by 0.03°C/yr under SSP2-4.5 and 0.07°C/yr under SSP5-8.5. This warming will augment the evapotranspiration rate which in turn will have a negative impact on the freshwater availability. Streamflow will increase by 5% under SSP2-4.5 and 9.49% under SSP5-85 which may increase sporadic flooding events. Climate change is expected to contribute to the deterioration of water quality shown by 61%, 36%, 79%, 115%, and 70% increased ammonia, chlorophyll-a, nitrite, nitrate, and phosphorus loadings, respectively, from 2022. The increase in temperature results in increases in nutrient loading and a decrease in dissolved oxygen. Overall, this research demonstrated the vulnerability of the catchment to climate change. The findings of this research can offer vital knowledge to policymakers on possible strategies for the sustainable management of water.

## 1. Introduction

Climate change is among the primary issues in sustainable water management [1]. According to studies and models of climate change at the global and regional scales, temperatures are rising, while precipitation is dropping and becoming more erratic and unpredictable [2, 3]. Climate change impacts water resources mainly through interception of the catchment hydrologic processes [4]. Research has previously demonstrated that aspects of climate change, such as variations in rainfall intensity and frequency, have an adverse effect on streamflow and the resulting storage capacity of catchments in Ethiopia [5]. Decrease in rainfall and increase in temperature affect the runoff depth and evapotranspiration and thus the catchment water level [6]. These changes manifest themselves in the form of increased intensity of floods or occurrence of severe droughts which severely affect the water resources at local and regional levels. As a result, the occurrences of extreme events like floods and droughts have increased in many Ethiopian towns [7]. Developing African countries including Ethiopia struggle to manage the risks related to climate change as a result of their low levels of infrastructure and human development. A combination of poor sanitation infrastructure and the accelerated urbanization results in 65% of the wastewater produced in Addis Ababa discharged straight into surface waters [8]. Lack of proper infrastructure under the changing climate has seen just 55% of the city served by the water supply service, while 50% receive less than twelve hours of service each day, and 25% do not even have formal service [9]. As a result, they suffer the greatest negative consequences of both climate variability and global environmental change. This could get worse if no action is made to reduce greenhouse gas emissions. This calls for a new holistic approach to sustainable water resource management.

UNDP [10] asserts that the results of climate change have the ability to reverse decades' worth of advancements in human development, particularly those made in the direction of the Millennium Development Goals (MDGs), and to jeopardize the achievement of the recently adopted Sustainable Development Goals (SDGs), such as SDG 6 on clean water and sanitation for all. Climate change modifies vital elements such as the hydrology of water resources, the water table, and changes in an area's precipitation patterns [11]. Sharp temperature increases are also likely to affect evapotranspiration and atmospheric water storage, which could alter rainfall quantities, frequency, and intensity as well as its seasonal and interannual variability and geographic distribution [12]. Reduced surface runoff, which affects surface and subsurface water flows, is also another way in which the increases in temperature affect groundwater recharge [13]. This has caused water stress for several countries.

Increased temperatures may also foster the growth of equipment-clogging algae, which gives water a bad taste and odor due to the development of bacteria and fungi [14]. Residents living adjacent to the Akaki catchment rivers often use their polluted water for residential purposes and other additional amenities. This may increase the prevalence of waterborne diseases. Decreased water quantities as a result of rise in temperatures coupled with population growth also result in unmet water demand in Addis Ababa city [15]. There is water rationing and residents currently go for many days or even weeks without water. Decline in water quality and water shortages can impact the operation of socioecological systems and health. Increased temperatures also result in reduced oxygen levels within the water [16]. In addition to having an immediate impact on stream temperature, higher air temperatures are predicted to increase evapotranspiration and possibly reduce water yield to rivers, which worsens the quality of the water because there will be less dilution [17]. Surface pollutants are transported with runoff; hence, the impact of climate change on runoff will also have a direct impact on how contaminants are transported and disposed of in aquatic ecosystems [18]. Understanding how climate change is affecting the stream water quality is therefore crucial for managing nonpoint source pollution for catchments.

Ethiopia has been suffering major effects of climate change for the past two to three decades [19, 20]. Heyi et al. [23] pointed out that climate change has a considerable impact on energy, water, and agricultural productivity. According to various research studies, the effects of climate change on the environment will only get worse in the future [22, 23]. Ethiopia has experienced rising temperatures and notable changes in rainfall [24]. The negative impacts of climate change have resulted in flooding in some places as a result of heavy rainfall, although droughts have also persisted in other areas because of inadequate rainfall [25]. Bhat et al. [26] reported that 73% of nitrogen load in a watershed was carried away with surface runoff during storm events. Increase in temperatures has been shown to result in increased water temperatures which reduce the CO_2_ levels in the water, hence increasing the pH and reducing the dissolved oxygen (DO) [27]. Temperature increase has also been shown to be positively correlated with nutrient loading in surface and groundwater such as increased nitrogen and phosphate concentrations [27, 28]. Drought events minimize the dilution capacity of streams, hence increasing the ammonium concentrations [27]. Changes in flow, water yield, and evapotranspiration determine freshwater availability. According to Liou and Mulualem [29], this impact has left many of Ethiopians vulnerable to severe food shortages and increased water insecurity. Therefore, assessing the impacts of climate change on water is critical for proper management and contextualization of the water balance at a local scale. The demand for water for domestic, industrial, agricultural, and residential uses rises with increase in urbanization and industrialization. Therefore, due to increased water use, urbanization, economic development, and climate variability, water scarcity issues are becoming more prevalent. The Gerfesa, Dire, and Legedadie reservoirs are part of the Akaki catchment as well as the main drinking water sources for Addis Ababa. As a primary source of water, the hydrological stability and water supply service function of the Akaki catchment hydrological is a cornerstone to the economic and social development of Addis Ababa city. The ongoing global climate change places additional constraints on the catchment's already inadequate water resources [15]. Due to the high variability of rainfall and high temperatures as a result of climate change, there is a significant stress on the water resources because of the reduction in water quality and quantity. This is exacerbated by anthropogenic activities in the city as a result of accelerated urbanization. Understanding the impact of climate change on the hydrology and water quality of the catchment could aid in addressing the water scarcity problem of Addis Ababa and its surroundings. However, the influence of climatic changes on water availability and water quality has gotten very little attention, despite the fact that academics have extensively studied its potential consequences on demand [30]. There has been little in-depth investigation of the watershed's ecosystem processes and landscape patterns. Therefore, a thorough approach to impact assessment needs to be adopted in order to analyze the probable impacts on hydrology, water quality, and ecology using process-based models of freshwater systems.

Using the SWAT modeling tool, the effects of climate change on the water supply and water quality in the Akaki catchment were assessed in this study. Studies across Africa have successfully used the SWAT model to investigate the effects of climate change and land use and land cover changes on water balance components and demonstrated its capability to generate hydrological processes with significant accuracy [31]. Other reasons that influenced the choice of the SWAT model for this study include the availability of input data for catchment modeling, acceptability, stability, and the computational efficiency of the model. The SWAT model is also the only free, semidistributed, physical-based model that can give all the hydrologic components and water quality parameters of interest. Climate model scenarios offer the most current knowledge for forecasting the potential impacts of climate change on the water quality and ecology of surface water bodies. Developing effective solutions to manage water resources requires a thorough comprehension of the root causes and consequences of the issues. Hence, the results of this study can be used by planners to develop sensible watershed management policies and mitigation plans.

## 2. Methodological Approach

### 2.1. Study Area

**Figure 1:**
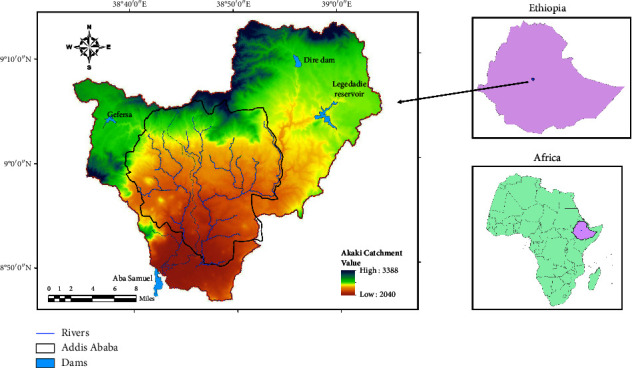
Location of the study area.

Akaki watershed is situated along the western edge of the major Ethiopian rift valley in central Ethiopia (Figure 1). The catchment is located at the northwestern Awash River [32]. The capital city, Addis Ababa, and smaller settlement villages are found in this catchment. The major tributaries of the catchment are the Great Akaki River in the east and Little Akaki River in the west of Addis Ababa. Addis Ababa is at the center of the catchment. The Akaki catchment covers an area of about 1445.40 km^2^ [33]. Both Little and Great Akaki rivers flow across the urban center of Addis Ababa towards Aba Samuel reservoir. From March through September are the seven major rainy months in the area, with June to September being the wettest months and lighter rainfall falling during the other months. The mean annual rainfall ranges between 1000 and 1,300 mm/yr, and the minimum and maximum mean annual temperatures are about 12°C and 24°C, respectively. The mean monthly temperature ranges between 7°C and 27°C. The highest maximum mean monthly temperatures are from the months of February to May, whereas the lowest maximum mean monthly temperatures are from July to September [33]. During the dry season, the streamflow, surface runoff, and infiltration rate might be lower because of the low rainfall and high temperatures recorded. Major land uses include residential and commercial settlements, planting of commercial trees, agriculture, and industries. A recent study by Gule et al. [34] indicates that built-up area is the most dominant land cover with over 85% coverage, while vegetated areas cover only 10.5% and agriculture 2.2%. Food processing industries, tannery and textile industries, leather processing industries, and construction are the most dominant industries concentrated along the Akaki catchment. Most agricultural land uses are located adjacent to water resources with the dominant crop being vegetables. Smallholder farmers use water from the Akaki catchment to irrigate their crops. Commercial farmers employ mostly surface irrigation and sprinklers, while subsistence farmers use traditional manual irrigation methods.

### 2.2. Major Datasets

**Figure 2:**
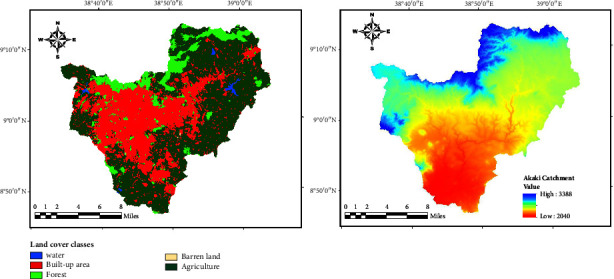
Land use map and digital elevation model covering the Addis Ababa city watershed.

In this work, the effects of climate shifts on catchment hydrology and water quality simulation were represented by the SWAT model. A variety of datasets, such as information on the soil, land use/land cover (LULC) map, climate, and digital elevation model data, were also used. These datasets came from several sources. The Ethiopian Meteorological Agency provided the climate data, which included information from its two meteorological stations, Bole and Obs, which serve catchments in Addis Ababa city. The metrological dataset from 1991 to 2021 contained time series information on daily precipitation (mm), relative humidity (%), hours of actual sunshine, minimum and maximum temperatures (°C), and wind speed (km/h). Before using the recorded meteorological data, missing data were filled in and data quality check was conducted. Inverse distance weighting was used to fill in the missing data as many studies have reported its accuracy. The consistency data from each individual station were then checked using the double mass curve technique. The digital elevation model (DEM) data were acquired from the U.S. Geological Survey Earth Explorer domain, https://earthexplorer.usgs.gov/, with a spatial resolution of 30°m. The Esri 2020 website, https://www.arcgis.com/home/item.html?id=d6642f8a4f6d4685a24ae2dc0c73d4ac, was used to obtain the LULC map. This map was created from Sentinel-2 imagery collected by the European Space Agency at a resolution of 10 meters. The LULC map and DEM map used in this study are depicted in Figure 2.

**Figure 3:**
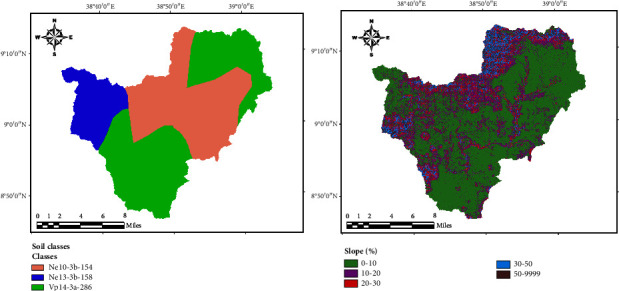
Soil map and slope map covering the watershed.

The hydrologic response of a catchment to the effects of climate change is influenced by the kinds and properties of the soil. Additionally, stream flow, sediment load, and nutrient content modeling are significantly impacted by the precision of soil data [35]. The source of the soil map was the Food and Agriculture Organization website, https://storage.googleapis.com/fao-maps-catalog-data/uuid/446ed430-8383-11db-b9b2-000d939bc5d8/resources/DSMW.zip. In the study region, *eutric nitosols* (Ne10-3b-154 and Ne13-3b-158) and *pellic vertisols* were discovered (Vp14-3a-286). For modeling purposes, a catchment has to be divided into several homogenous subbasins or hydrologic response units which have distinct soil, slope, and land use properties. Therefore, the slope was divided into 5 classes; 0–10%, 10–20%, 20–30%, 30–50%, and >50%.  Figure 3 shows the soil and slope map of the study area.

The Ethiopian Ministry of Water Resources and Energy provided stream flow data for the flow monitoring stations at Akaki, Little Akaki/Gefersa, and Mutinicha/Legedadi from the years 1990–2021. The quality of the measured streamflow data was assessed by visual inspection and accumulated plots in Excel before applying it to the calibration and validation of the SWAT model to ensure that there are no gaps and unrealistic peaks in the data series. Missing data were filled using the Markov chain Monte Carlo (MCMC) approach using multiple imputation algorithms in XLSTAT.  Table 1 includes a list of the sources of these input data.

Four climate models were acquired from https://esgf-node.llnl.gov/search/cmip6/ for daily precipitation and minimum and maximum temperature data from historical (1991–2014) and future (2040–2099) periods.  Table 2 shows the four selected climate models based on their resolution and previous studies in the subject. These models were also chosen because they performed well in the study area and provided quality information. The four CMIP6-GCMs under SSP2-4.5 and SSP5-8.5 scenarios were used to produce climate simulation data (*T*_min_, T_max_, and rainfall). CMhyd software was then used to statistically downscale the acquired climate data and bias-correct it using the distribution mapping method. An ensemble of the four models was then made. Due to boundary conditions, inherent unpredictability, and variations in model design, climate models contain a great deal of uncertainty. Mean ensemble models have been proven to provide an overall best comparison to observed climate data. Therefore, the simple arithmetic mean method was used to make the multimodel ensemble of the four CMIP6-GCMs by aggregating the rainfall, *T*_max_, and *T*_min_ for each year for all the three models.

### 2.3. Climate Downscaling and Bias Correction

Downscaling was performed prior to applying the GCMs-CMIP6 data to the hydrological model [36]. The projected future temperature and rainfall over the catchment were downscaled using a statistical downscaling method because it is simpler and easy to use in analyzing the effects of climate change at the local scale. The CMhyd tool was used to downscale large-scale historical and future climate data from CMIP6 models with SSP4.5 and SSP8.5 scenarios [37].

Climate model predictions for temperature and precipitation typically do not match the statistical characteristics of the observed time series data, which can lead to erroneous conclusions when applied without correction, for instance, if the amount of rainfall and its intensities are not precisely recorded and extreme temperatures are underestimated. Therefore, bias corrections were applied in this study to minimize the bias in climate model output data [37]. Upon the extraction and downscaling of climate data, bias correction was performed using the distribution mapping method in the CMhyd tool. The distribution mapping approach is used to align and adjust the data outputs from climate models with the observed data. It is predicated on the notion that the observed and simulated climate data follow a particular frequency distribution. As shown in Table 2, the climatic data from the four chosen GCM CMIP6 models were downscaled and bias-corrected using the rainfall and temperature data from the two stations. An ensemble model was constructed using the results of downscaling and bias correction.

### 2.4. Performance of Climate Models

Using the downscaled and bias-corrected monthly historical data (rainfall, *T*_min_, and *T*_max_) and monthly observed rainfall, *T*_min_, and *T*_max_ from 1991 to 2014, the multimodel ensemble mean's performance was investigated. Four statistical metrics, including the correlation coefficient (*r*), root mean square (RMSE), percent bias (PBIAS), and Kling-Gupta efficiency (KGE), were validated using RStudio, and Excel.r conveys the relationship between the observed data and climate model data and ranges from −1 to 1, with 1 indicating a perfect relationship. RMSE predicts the potential error and PBIAS measures the variations within the simulated climate model data. Both RMSE and PBIAS range from 0 to infinite with the best value being 0. KGE evaluates the different properties of the ensemble model and ranges from 1 to infinite, with 1 being the best value.

### 2.5. SWAT Model Setup, Calibration, Validation, and Sensitivity Analysis

The Soil and Water Assessment Tool (SWAT) model was used to generate the hydrologic model. The ArcSWAT 2012 application, which is an ArcGIS interface, was used for this. Using the DEM and stream network data in ArcSWAT, the area was divided into 132 hydrological response units (HRUs) and then delimited into 5 subbasins with manually specified outlets based on the variation in land use, soil type, and slope. Parameters of SWAT models were varied at different spatial levels: HRUs, subbasins, and basin [38]. Calibration and validation were conducted via the interface of the SWAT-CUP tool using the Sequential Uncertainty version2 (SUFI-2). Twenty-four hydrological parameters with default upper and lower bounds were chosen for global sensitivity analysis from the SWAT-CUP based on references from other literature [33, 39]. These parameters were chosen because of their role as external factors that can influence hydrological processes such as streamflow and water quality. Using multiple regression methods with Latin hypercube parameters of the objective function, t-statistics, and *P* values, the sensitivity parameters were detected after running 2000 simulations. High t-statistic values and small *P* values close to zero demonstrate a sensitive parameter [40]. To calibrate and validate the SWAT model, observed hydroclimatic data (rainfall, temperature, and streamflow) from 1990 to 2014 were divided into three stages. The model was initialized using data from 1990 to 1993, calibrated using data from 1994 to 2004, and validated using data from 2005 to 2013. The SWAT model was then calibrated again using parameters for which it demonstrated great sensitivity.

To compare simulated and observed data, the model's performance was assessed using the coefficient of determination (*R*^2^), Nash–Sutcliffe Efficiency (NSE), and percent bias (PBIAS) [41]. *R*^2^ has to be between 0 and 1 with higher values indicating a better prediction. According to Moriasi et al. [41], the NSE value should exceed 0.5 to be able to judge hydrological calibration and validation as satisfactory. PBIAS estimates the percentage trend of simulated data in relation to observed data with positive values indicating overestimation and negative values indicating underestimation. A PBIAS of 0 indicates optimal performance of the hydrological model, where values less than 0.1–0.15 (10%–15%) are considered very good performance. KGE indicates the relationship between observed climate data and simulated climate data values. It ranges between −∞ and 1, where 1 indicates a perfect fit.

### 2.6. Homogeneity Test and Trend Analysis

A test for change point identification is a crucial method to investigate the time interval during which a notable shift occurred in the time series of variables. The homogeneity test and trend analysis were used to identify breaks or change points as well as trends in rainfall and temperature. To create the annual time series, the ensemble means of maximum and minimum temperatures and rainfall were combined. To find the most likely period for a break in an annual time series of data, the Buishand range test, standard normal homogeneity test (SNHT), and Pettitt's test were applied. There are no presumptions regarding the distribution of rainfall and temperature data when using any of these three nonparametric change point techniques. They provide a signal for when variations in the average temperature and precipitation happen [42]. Homogeneity was examined at a 5% significance level using XLSTAT. Decisions on the possibility of change points were made based on the criteria outlined by Ilori and Ajayi [42], who described that the entire data series is split into two subseries where there is a discernible shift in the data (change point). Temperature and rainfall trend assessments were performed for both historical and future periods, with historical (1990–2014), midfuture (2040–2069), and far-future (2070–2099) under SSP2-4.5 and SSP5-8.5 scenarios. The baseline period was chosen based on data availability and then future periods based on the baseline period and guidelines of appropriate gap between baseline and future period for the SWAT model when using CMIP 6 climate model data. The data were divided into two periods after breakpoint detection, and trend analysis was then carried out. A Mann–Kendall nonparametric test [43] was employed to ascertain the direction of changes in time series. The Mann–Kendall test is a nonparametric test that detects whether there are trends in the climate data series and works best with independent data but is less sensitive to outliers. The statistical value (*P* value) was applied to test the null hypothesis with a 5% level of confidence. The magnitude of the trend was calculated using Sen's slope estimator. While a negative score indicates a downward trend, a positive value suggests an upward trend. Sen's slope can be estimated using the following equation:

(1) $$\begin{array}{c} S=\overset{n}{\underset{i=1}{\sum }}\left\{\overset{n}{\underset{j=i+1}{\sum }}\text{sgn}\left(\text{Ri}-\text{Rj}\right)\right\}, \end{array}$$

where sgn (*x*) = 1 for *x* > 0, sgn (*x*) = 0 for *x* = 0, and sgn (*x*) = −1 for *x* < 0.

### 2.7. Evaluation of Climate Change Impacts on Water Quality and Hydrology

The SWAT model was used to evaluate the climate change impacts on the hydrology and water quality over two time horizons for both SSP2-4.5 and SSP5-8.5 scenarios. The validated SWAT model was run using the multimodel ensemble mean of rainfall, *T*_max_, and *T*_min_. The baseline period was analyzed using mean annual and monthly streamflow and water quality data to evaluate the relationship between different hydrological components and water quality parameters in the catchment. To detect the effects of climate change on the hydrology and water quality in the Akaki catchment, a comparison of yearly and monthly hydrological components and water quality parameters from both periods and the baseline was performed.

## 3. Results

### 3.1. Multimodel Ensemble Climate Projection Evaluation

The performance of the multimodel ensemble mean was evaluated by comparing monthly simulated historical data with monthly observed historical data for rainfall, *T*_max_, and *T*_min_ at all stations.  Table 3 shows the multimodel ensemble mean for monthly observed rainfall, maximum temperature (*T*_max_), and minimum temperature (*T*_min_) over all stations, with *r*-value ranging between 0.76 and 0.91, indicating a strong correlation between the observed data and the climate model simulated data. RMSE ranged between 0.91 and 2.31 mm, and 0.5 and 0.79, respectively, indicating that there is no much difference between the predicted climate data and the actual observed data. KGE was above 0.5, indicating the accuracy of the model. Therefore, the multimodel ensemble mean data agreed with the observed data at all stations, with PBIAS values ranging from −1.9% to 0%, indicating a good estimation of rainfall, *T*_min_, and *T*_max_ by the ensemble model. These metrics indicate that the multimodel ensemble mean performed well, with better agreement between observed and simulated rainfall, *T*_min_, and *T*_max_ values at the two stations across the watershed. This implies that the climate model data provide a good estimation of the climate in the study area and therefore can be used to predict the impact of climate change on the catchment hydrology and water quality.

### 3.2. Homogeneity of Future Climate Variables

The homogeneity of rainfall, *T*_min_, and *T*_max_ at all stations was investigated under SSP2-4.5 and SSP5-8.5 scenarios using XLSTAT. The results demonstrated the inhomogeneity of rainfall and temperature data between 2040 and 2099. Under SSP2-4.5, the annual rainfall series had change points at 2073 for station 1 and 2057 for station 2. For *T*_max_, the change point was found at 2070 in all stations, while for *T*_min_, change points were found at 2062 and 2069 in all stations under SSP2-4.5 scenario. Under the SSP5-8.5 scenario of the annual rainfall, change points were detected in the year 2077 for both meteorological stations. *T*_min_ results indicated a break at 2068 for both stations, while *T*_max_ showed change points at the years 2067 and 2071 under the SSP5-8.5 scenario. The change points in future rainfall, *T*_max_, and *T*_min_ data indicate that there will be a shift in temperatures and rainfall in the future under both scenarios.

### 3.3. The Trend Analysis of Historical and Future Climate Variables

The rainfall and temperature data were evaluated monthly and annually in separate time intervals: historical (1991–2014) and future (2040–2069 and 2070–2099) under both SSP2-4.5 and SSP5-8.5 emission scenarios.

#### 3.3.1. Historical Annual Rainfall and Temperature Trends

The Mann–Kendall trend test and Sen's slope estimator results for historical rainfall, *T*_min_, and *T*_max_ are shown in Table 4. The Mann–Kendall trend test for mean annual rainfall demonstrated an insignificant upward trend in both stations under certain conditions of ensemble mean climate models. The historical data showed statistically significant rising trends for maximum and minimum temperatures, with the rates ranging from 0.016 to 0.058°C per year. The observed and simulated slopes had identical rising trends, demonstrating the value of downscaled *T*_max_ and *T*_min_ data for trend analysis. Trend analysis is important for showing changes in patterns of rainfall and temperatures in the future. Increases in rainfall and temperature trends imply a shift in climate conditions in the area, which in turn might impact water quality and availability.

#### 3.3.2. Future Annual Rainfall and Temperature Trends

The SSP2-4.5 and SSP5-8.5 scenarios were used to evaluate potential changes in rainfall, *T*_max_, and *T*_min_ for the midfuture (2040–2069) and far-future (2070–2099) periods.  Table 5 displays the rainfall trend analysis for the two scenarios from 2040 to 2069. The annual mean rainfall increased insignificantly under the SSP5-8.5 scenario for all stations and insignificantly for Bole station under SSP2-4.5 at a 5% significant level. Sen's slope estimator anticipated a minor rise and fall of 1.410 and 19.39 mm/year and −0.99 and 12.8 mm/year, under SSP2-4.5 and SSP5-8.5, respectively. *T*_max_ multimodel ensemble mean trend results for the SSP2-4.5 and SSP5-8.5 scenarios showed that, at the 5% level of significance, both the SSP2-4.5 and SSP5-8.5 scenarios resulted in significant increases of 0.03 and 0.014°C/yr for SSP2-4.5 and 0.04 and 0.03°C/yr under SSP5-8.5. For *T*_min_, a significant increase of 0.03 and 0.02°C/yr was noted under SSP2-4.5 and increases of 0.07 and 0.05°C/yr trends were noted under SSP5-8.5 across all stations shown in Table 5. These results imply that climate will shift in the future, with increases in rainfall and temperatures, exposing communities to alternating inundations and droughts. This clearly indicates that climate is changing within the study area which will increase uncertainties related to drought, severe storms, rainfall frequency, number of rainy days, and intensity all of which will have an impact on water in the catchment.

### 3.4. Hydrological Model Evaluation

#### 3.4.1. Hydrological Model Sensitivity Analysis, Calibration, and Validation

Sensitivity analysis was employed to determine the key parameters influencing streamflow and water quality in the SWAT model [44]. Monthly streamflow data from 1994 to 2004 and 2005 to 2013 were used during the calibration and validation phases, respectively. In a global sensitivity analysis of 24 streamflow and water quality parameters, 11 parameters were demonstrated to be flow-sensitive based on their t-statistic and *P* value. These included surface runoff processes parameters (CN_2_), ground-water parameters (GWQMN, RCHRG_DP, GW_DELAY, ALPHA_BF, and GW_REVAP), lateral flow process parameters (HRU_SLP), and nutrient concentration sensitive parameters (SHALLST_N, N_PERCO, PSP, and PPERCO). RCHRG_DP.gw, GWQMN.gw, HRU_SLP.hru, GW_DELAY.gw, and CN2.mgt were the five most sensitive parameters (Table 6). The parameters indicate an impact on the catchment hydrological processes such as streamflow, percolation, and groundwater recharge. The sensitivity of parameter CN_2_ indicated that the characteristics of the catchment are significantly affecting surface runoff. The parameters of nitrate and phosphorus movement have a larger influence on nutrient concentration indicating the impact on water quality. To improve the model's performance during calibration, the selected parameters were iteratively modified within a reasonable range until an acceptable agreement between observed and simulated streamflow output was found. After calibration, validation was performed using the same set of calibrated flow parameters. The calibrated and validated parameters are shown in Table 6 along with the fitted values.

The calibration and validation procedures included comparisons of the recorded monthly mean streamflow at the Akaki catchment's outlet with its modeled discharge values. The statistical indices of the calibration and validation findings, as well as the P-factor and R-factor, were obtained. P-factor and R-factor indices are used to evaluate the strength of the calibration and validation. R-factor provides a measure of sensitivity where an ideal has to be less than 1.5 [40]. P-factor determines the significance of sensitivity of the parameters and varies from 0 to 1, where 1 indicates a perfect model simulation considering the uncertainty. During calibration, the P-factor and R-factor were 0.96 and 0.83, respectively. For validation, the P-factor and R-factor were 0.90 and 0.61. According to [40], these P-factor and R-factor values found in this study were within the standard. The performance indices during the calibration and validation period also indicated very good results [41]. The results of this study showed that the value of NSE, *R*^2^, PBIAS, and KGE for the calibration period was 0.88, 0.89, −1.8, and 0.94, respectively. For the validation period, NSE, *R*^2^, PBIAS, and KGE were 0.96, 0.95, −0.9, and 0.99, respectively, all of which indicate that the model is accurate. When the *R*^2^ and NSE values for stream flow are greater than 0.5, the model performance is deemed satisfactory [41]. Low absolute values for PBIAS imply better simulations, and zero is the ideal value for accurate model prediction. KGE of 1 is deemed a perfect fit [41].

### 3.5. Hydrologic Impact Assessment

**Figure 4:**
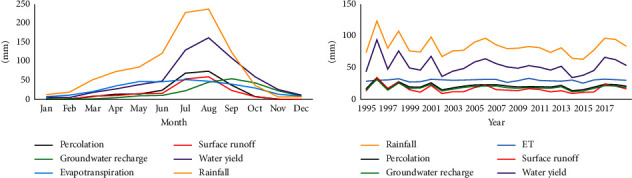
Historical monthly and annual hydrological components.

The impact of climate change on the hydrology in the catchment was evaluated for the baseline period for monthly and annual climate conditions. Evaluated parameters included rainfall, evapotranspiration, percolation, water yield, surface runoff, and groundwater recharge which are essential parameters predicted by the SWAT model for adequate water management and planning in the study area (Figure 4).

**Figure 5:**
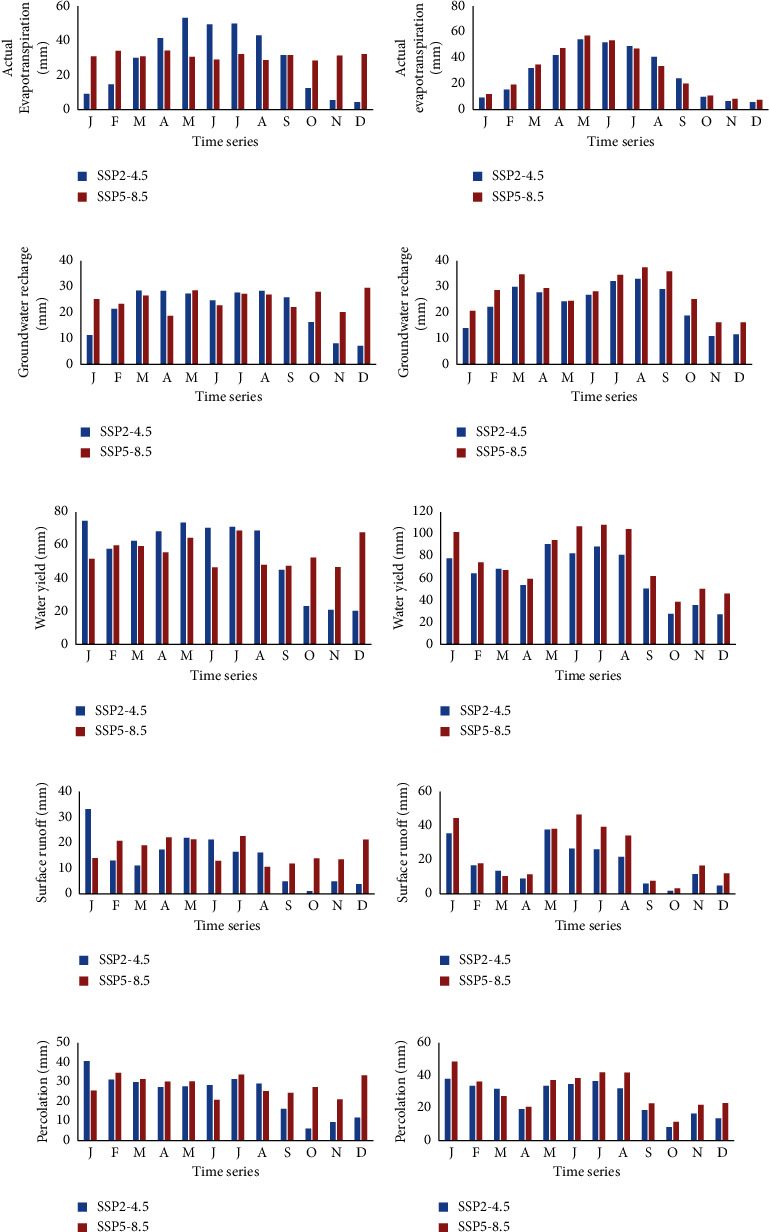
Projected mean monthly hydrological components in the catchment under SSP2-4.5 and SSP5-8.5 scenarios for both mid and far future. (a, c, e, g, i) 2040–2069. (b, d, f, h, j) 2070–2099.

The SWAT model was run to evaluate the climate change impact on hydrological components including surface runoff evapotranspiration (ET), streamflow, groundwater recharge, water yield, and percolation.  Figure 5 summarizes the monthly future predicted hydrologic components for the SSP2-4.5 and SSP5-8.5 scenarios at the catchment outlet. Similar hydrological behavior was detected between the two scenarios in both mid- and far-future periods. Future actual evapotranspiration increased from April to August, with the maximum possible actual evapotranspiration recorded in May for both scenarios (Figures 5(a) and 5(b)). Changes in evapotranspiration may negatively affect water availability and ecosystem health of the catchment. Groundwater recharge showed a constant variation throughout the months (Figures 5(c) and 5(d)). The mean monthly projected water yield generated by the SWAT model in midfuture will slightly increase under the SSP2-4.5 scenario from February to August and then start decreasing in September under both scenarios. In the far-future (2070–2099), the monthly water yield will significantly decrease under SSP2-4.5 and SSP5-8.5 scenarios compared to the midfuture period (Figures 5(e) and 5(f)). A decrease in water yield will also result in a decrease in the streamflow, water availability, and groundwater recharge. The months of January and May–August recorded the highest values of surface runoff under both scenarios for mid- and far-future periods (Figures 5(g) and 5(h)), which means that the groundwater recharge and surface runoff will also increase during these months. The mean monthly simulated percolation generated by the SWAT model indicated a fluctuating trend in the future under both SSP2-4.5 and SSP5-8.5 scenarios (Figures 5(i) and 5(j)).

**Figure 6:**
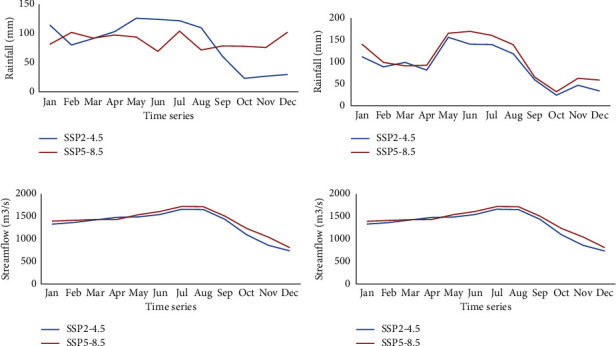
Future monthly rainfall and streamflow in the catchment under the SSP2-4.5 and SSP5-8.5 scenarios. (a, c) 2040–2069. (b, d) 2070–2099.

Figures 6(a) and 6(b) show the mean monthly rainfall for future periods under future scenarios. The results revealed that the maximum rainfall will be in May-August in both periods under the two scenarios. Figures 6(c) and 6(d) show the anticipated streamflow for midfuture (2040–2069) and far-future (2070–2099) periods under both SSP2-4.5 and SSP5-8.5 scenarios. The midfuture mean monthly streamflow analysis revealed that streamflow will peak in July (1659.73 m^3^/s) for SSP2-4.5 and (1721.03 m^3^/s) for SSP5-8.5 scenarios, while the minimal mean monthly streamflow for SSP2-4.5 (734.09 m^3^/s) and SSP2-8.5 (809.60 m^3^/s) will be in December.  Figure 6(d) shows statistical results on mean monthly streamflow in the far-future period (2070–2099), which, like the midfuture period demonstrated that streamflow peaked from July-August with a maximum of 1726.03 m^3^/s under SP2–4.5 and 1778.47 m^3^/s under SSP5-8.5.

### 3.6. Water Quality Impact Assessment

**Figure 7:**
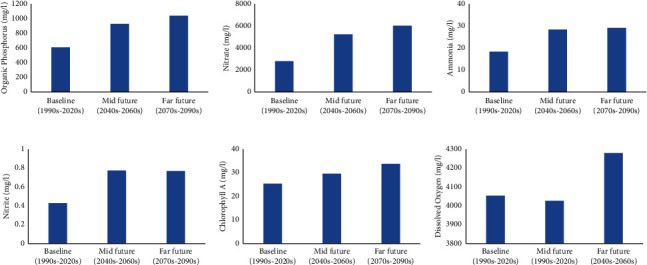
Effects of climate change on water quality parameters over the baseline period, midfuture, and far-future periods: (a) changes in organic phosphorus loadings, (b) changes in nitrate concentration, (c) changes in ammonium content, (d) changes in nitrite concentration, (e) changes in chlorophyll A content, and (f) changes in dissolved oxygen.

Results from the SWAT model output showed that the will be an increase in the future organic phosphorus, nitrate, ammonia, chlorophyll-*a*, and nitrite in the water (Figures 7(a)–7(e)). Phosphorus loading will increase by 70% from 608.56 to 1038.18 mg/l. Nitrate will increase from a concentration of 2805.16 mg/l during the baseline period to 6022.69 mg/l by future, an increase of 115%. Ammonia will increase from 18 mg/l to 29 mg/l, an increase of 61%. Nitrite concentration will increase by 79% from 0.43 to 0.77 mg/1, while chlorophyll A will increase by 36% from 25 to 34 mg/l. This means that the water quality in the catchment areas will continue to deteriorate in the mid- and far-future periods. High nutrient concentration water will negatively impact human health, and the aquatic ecosystem will also result in increased algal growth within the catchment. Dissolved oxygen (DO) is not expected to change much between the baseline period and the midfuture period, but by the far-future period, it is expected to show a sharp increase from 4054 to 4280 mg/l, an increase of 5.6% (Figure 7(f)). High DO in water is preferred because it improves the quality of drinking water by oxidizing organic matter that would have otherwise created undesirable taste of the drinking water. Conversely, where metallic pipelines are used, corrosion of the metal could make the water source of health hazards. This can cause problem with the urban water supply infrastructure of the city. Also, very high levels of DO can lead to supersaturation which is responsible for gas bubble disease in fish and invertebrates.

## 4. Discussion

Under the SSP2-4.5 and SSP5-8.5 scenarios, the predicted mean annual rainfall trend analysis revealed insignificant increases and an erratic trend in both periods. According to this result, under the SSP2-4.5 and SSP5-8.5 scenarios, the catchment's peak rainfall will shift from July to September to a longer period of May to September in the midfuture (2040–2069). Increased variability in rainfall may decrease groundwater recharge in the catchment area because more frequent heavy rainfall will affect the infiltration capacity of the soil, thereby increasing surface runoff. This is due to the fact that only intense downpours can penetrate quickly enough before evaporating. Variations in rainfall will also contribute to changes in streamflow [45]. The data gathered in this study suggests rising temperature patterns that are consistent with research from other studies in Ethiopia [46]. Katipoğlu [47] also reported an increase in temperatures from the Euphrates basin and Bursa and a shift in monthly temperature and rainfall trends. This can be attributed to the infrastructure expansion in Addis Ababa city which lies within the catchment. This is because increased imperviousness contributes to the warming of the ground surfaces. The availability of freshwater and agricultural production will probably be negatively impacted by this rise in future temperatures [31]. Higher evapotranspiration rates lead to reduction in surface runoff, soil moisture, and groundwater recharge, and as a consequence, lesser and lesser amounts of water will be available in the catchment [48]. Since most of the population depends on rain-fed and irrigated agriculture, the productivity will reduce leading to food insecurity. Decreased water availability also implies that climate change will have a negative impact on the socioeconomic status of Addis Ababa city. As a result, effective interventions are crucial to ensuring sustainability and water security. Moreover, the findings of this study are important considering that with fluctuating rainfall and increasing temperatures, there may be corresponding increases in drought and sporadic flood events in the basin in the 21st century [49].

From the results, it appears that the effects of the two scenarios will result in drastic changes for the Akaki catchment hydrologic regime. When compared to the historical period (1991–2018), actual evapotranspiration will increase the future under the SSP2-4.5 and SSP5-8.5 scenarios. This increase in evapotranspiration is due to anticipated temperature increases in the twenty-first century. An interesting case is modeled for the wet season, especially April to August, where a significant increase in runoff is formed. This study is in agreement with similar studies which suggested that a shift in seasons will likely happen due to projected changes [50]. Gule et al. [51] estimated that the built-up area in Addis Ababa city has increased by over 338% from the period of 1991 to 2021. This increase in the built-up area comes with increased impervious areas which in turn limits the seepage of water into the ground. According to some studies, a 10% increase in imperviousness can result in an increase in surface runoff since it reduces infiltration, hence altering natural hydrological systems and resulting in frequent floods [52]. Consequently, a particularly significant rise in runoff will result from the anticipated increase in precipitation and infrastructure expansion in the Akaki catchment. Some explanation could be provided by the distribution of surface and groundwater flow. A key for the long-term planning and management of the water resources in a watershed, considering future changes in the patterns of the climate, water demand, and water availability, is not only the possible changes in the annual hydrologic components under climate change but also the possible changes in the seasonal hydrologic components [53].

On the other hand, phosphorus, nitrate, nitrite, ammonia, and chlorophyll-*a* showed a tendency to increase in mid- and far-future periods. This is in agreement with findings by Gule et al. [34] who found that nutrient loadings will continue to increase in Addis Ababa surface water and that the water quality will continue to deteriorate over time. This might be due to the increased surface runoff from increased rainfall and increased impervious areas around the catchment. The water quality of the rivers within the Akaki catchment is poor because of industrialization and accelerated urbanization of Addis Ababa city which lies within the bound of the catchment [34]. Anthropogenic activities in close proximity to water sources have impacts on the water quality since land and water ecosystems are connected by surface runoff, stream networks, and groundwater systems. Hence, cultivation in close proximity to water limits the inherent ability of wetland to act as buffers, hence reducing their capacity to absorb and store surplus nutrients and to filter sediments. Deforestation and other factors, such as the presence of agriculture adjacent to water resources, can affect the overall water quality by increasing sedimentation and nutrient additions in waterbodies. For instance, a study by Gule et al. [34] revealed that turbidity has a highly substantial positive association with agriculture and built-up area. This implies that the water becomes increasingly turbid as the amount of built-up area increases. Ammonia, nitrite, and nitrate concentrations are also affected by increases in the built-up area and a reduction in vegetated areas and bare land [34].

Most of the population tends to reside in Addis Ababa city, resulting in pollution loads from agriculture and waste. Since Addis Ababa does not have proper sewage connection lines, most of the household and industrial wastes are directly dumped into the rivers. Decline in water quality and water shortages can impact the operation of socioecological systems and health. A large fluctuation in the river regime coefficients (the ratio of maximum flow to minimum flow ranging from 1659.73 m^3^/s to 734 m^3^/s) results in difficulties in supplying water, controlling floods, and managing water quality [54]. Low flows during the dry season may lead to an increase in the pollution level. By changing nutrient flows into water sources, anthropogenic activities have impacted the water quality. According to Gule et al. [34], ammonia, nitrite, and nitrate concentrations are affected by changes in the amount of bare land and built-up area in Addis Ababa city. The nitrate concentrations' positive relationships with the built-up area and agriculture suggested that these land use/land cover alterations were the primary sources of nitrate in the city's water supplies [34]. In light of this, decision-makers in the watershed need quantitative information to develop adaptation strategies against climate change. The city residents are the main actor in the policy's execution; hence, a frequent awareness campaign with their participation is important. Since most water resource developments are conducted at the local level, studies such as this, which concentrate on the potential future implications of climate change on water supplies' catchment at the local level, are crucial. Prior to recommending adaptation strategies that can lessen the harm that climate change and upcoming developments can do to water resources, it is necessary to evaluate the extent of the impact. Water managers must quantify and evaluate the danger that climate change may bring about in order to take proactive steps towards risk reduction and climate adaptation. As a result, the findings of the research will provide comprehensive and practical knowledge for managing water resources more effectively and putting climate change mitigation strategies into action in Addis Ababa.

## 5. Conclusions and Recommendations

The study's findings demonstrate that changes in temperature and precipitation, regardless of their magnitude, timing, or both, have a major impact on the quantity and quality of water in the Akaki catchment. In order to support more predictable water demand and sustainable water availability, the annual change and seasonal variation of hydrological components due to future temperature increase and precipitation changes should be evaluated and incorporated into water resource planning and management [55]. If no mitigation actions are taken, climate change may result in an increase in the amount of nutrients entering rivers by altering the growth season of crops, fertilization practices, and human activities. Because most global challenges including food security, biodiversity loss, water security, and human health are linked to extreme events brought on by climate change, it is crucial to comprehend the pattern of precipitation and temperature trends as well as their variability [56]. Therefore, the results of this study will be useful to basin planners, policymakers, and water resources managers in developing adaptation strategies to offset the adverse effects of climate change on water resources [57]. The study was limited in that only a few water quality parameters were assessed; therefore, it is recommended that future studies add analysis of climate change impact on more microbiological and physicochemical parameters. The SWAT model also relies on empirical formulas. This limitation was overcome in this study by comparing the observed data from different institutions with literature as well. However, it is recommended that future studies compare the results of the SWAT model with other models to increase accuracy. Even though the effects of climate change on the hydrology and water quality of the catchment have been effectively assessed, future research should concentrate on determining which factor between land use and climate change has a greater influence on changes in hydrology and water quality in order to prioritize mitigation and adaptation measures. Future research should also evaluate the CMIP5 and CMIP6 model datasets and compare how well they can reproduce the temperature and rainfall spatiotemporal patterns in the Akaki catchment.

## Figures and Tables

**Table 1:** Basic input data for the SWAT model.

Data type	Data sources
DEM	U.S. Geological Survey's (USGS) Earth Explorer domain
Soil map	Food and Agriculture Organization (FAO) website
Land cover map	Esri 2020 Land Cover (mature support) website
Climate data	Meteorological Agency of Ethiopia
Discharge data	Ethiopian Ministry of Water Resources and Energy
Water quality data	Addis Ababa Water and Sewerage Authority
Land management practices	Field investigation

**Table 2:** Selected CMIP6 global climate models in this study.

Model	Institution/country	Resolution in (°)
GFDL-ESM4	Geophysical Fluid Dynamics Laboratory, NJ, USA	1.0 × 1.25
INM-CM5-0	Institute for Numerical Mathematics (INM), Russian Academy of Science, Moscow, Russia	2.0 × 1.5
MIROC6	Japan Agency for Marine-Earth Science and Technology (JAMSTEC)	1.4 × 1.4
MRI-ESM2-0	Meteorological Research Institute, Ibaraki, Japan	1.1 × 1.1

**Table 3:** Monthly rainfall, *T*_max_, and *T*_min_ statistical performance metrics from 1990 to 2014 for the multimodel ensemble mean over the watershed.

Station	Metrics	*r*	RMSE	PBIAS	KGE
1	Rainfall	0.79	2.04	−1.9	0.72
	*T*_max_	0.83	1.47	0	0.74
	*T*_min_	0.8	0.91	0	0.77

2	Rainfall	0.76	1.21	−0.55	0.77
	*T*_max_	0.91	2.31	0	0.5
	*T*_min_	0.77	1.03	0	0.7

**Table 4:** Mann–Kendall and Sen's slope estimator value for annual rainfall and maximum and minimum temperatures in the watershed from 1991 to 2014.

	Rainfall		Maximum temperature		Minimum temperature	
	Obs	Bole	Obs	Bole	Obs	Bole
MK trend	0.022	0.145	0.370	0.42	0.572	0.58
*P* value	0.903	0.338	0.011^*∗*^	0.004^*∗*^	<0.0001^*∗*^	<0.0001^*∗*^
Sen's slope	0.441	10.24	0.025	0.016	0.058	0.044

With ^*∗*^ referring to significantly increasing or decreasing trends.

**Table 5:** Projected Mann–Kendall trend and Sens's slope estimator results for annual rainfall for the two meteorological stations within Addis Ababa for the midterm period (2040–2069).

		SSP2-4.5			SSP5-8.5		
		MK trend	*P* value	Sen's slope	MK trend	*P* value	Sen's slope
Rainfall	Obs	0.03	0.86	1.410	−0.03	0.86	−0.99
	Bole	0.30	0.02^*∗*^	19.39	0.241	0.06	12.8

*T*_max_	Obs	0.45	0.0003^*∗*^	0.03	0.65	3.03*E* − 08^*∗*^	0.04
	Bole	0.41	0.001^*∗*^	0.014	0.6	7.01*E* − 07^*∗*^	0.03

*T*_min_	Obs	0.52	2.47*E* − 05^*∗*^	0.03	0.77	0^*∗*^	0.07
	Bole	0.51	4.28*E* − 05^*∗*^	0.02	0.73	0^*∗*^	0.05

Where ^*∗*^ signifies increasing or decreasing trends.

**Table 6:** Calibrated parameters and their fitted values.

Parameters	Minimum value	Maximum value	Fitted values
v_RCHRG_DP.gw	0	0.558	0.097
v_GWQMN.gw	1774.31	5000	3067
r_HRU_SLP.hru	0.27	0.81	0.70
v_GW_DELAY.gw	165.93	497.82	299.60
r_CN2.mgt	−0.31	0.03	−0.18
v_GW_REVAP.gw	0.068	0.16	0.13
v_SHALLST_N.gw	0	0.62	0.49
v_PPERCO.bsn	10	15.30	14.25
v_PSP.bsn	0.42	1	0.88
v_ALPHA_BF.gw	0.47	1	0.68
v_NPERCO.bsn	0.40	1	0.42

## Data Availability

All relevant data have been included in the manuscript, and if more data are required, they will be made available on request.
